# Design of the performance outcome scoring template (POS-T) with example application on CO_2_ emissions reduction amongst 36 OECD member countries

**DOI:** 10.1038/s41598-022-08368-w

**Published:** 2022-03-15

**Authors:** Benjamin P. Raysmith, Toomas Timpka, Jenny Jacobsson, Michael K. Drew, Örjan Dahlström

**Affiliations:** 1 Athletics Research Centre, Linköping University, Linköping, Sweden; 2 Western Australian Institute of Sport, Perth, Australia; 3 Athletics Australia, Melbourne, Australia; 4 Swedish Athletics Association, Stockholm, Sweden; 5 Athlete Availability Program, Australian Institute of Sport, Bruce, ACT Australia; 6 Australian Collaboration for Research into Injury in Sport and Its Prevention (ACRISP), Perth, Australia; 7 University of Canberra Research Institute for Sport and Exercise (UCRISE), Canberra, ACT Australia; 8 Department of Behavioural Sciences and Learning, Linköping University, Linköping, Sweden

**Keywords:** Statistics, Software, Climate change

## Abstract

In applied program settings, such as in natural environment control and education, performance evaluation is usually conducted by evaluators considering both self-comparison and comparison with peers. We have developed the performance outcome scoring template (POS-T) for assessments with high face-validity in these settings. POS-T puts achievements of individuals or groups in context, i.e. the resulting performance outcome score (POS) reflects a meaningful measure of performance magnitude with regards to internal and external comparisons. Development of a POS is performed in four steps supported by a statistical framework. Software is supplied for creation of scoring applications in different performance evaluation settings. We demonstrate the POS-T by evaluation of CO_2_ emissions reduction amongst 36 OECD member countries.

## Introduction

Performance evaluation seeks to examine the achievement of predetermined objectives or goals by individuals or groups through the broad assessment of processes (inputs and activities) and results (outputs and outcomes)^1–3^. These evaluations are used to assess program efficiency and effectiveness and provide accountability to resource allocation, strategy and policy direction^3–5^. Performance evaluations are usually case-specific and defined by the stakeholders with the authority and responsibility to do so^6,7^. Furthermore, they are expected to provide a contextual judgement of performance at a moment in time and require the measurement of credible ongoing outputs (performance measures) that relate specifically to the needs of the evaluators^3,8–10^. The assessment of goal achievement lies with the process of performance evaluation and considers a broad array of factors that include addressing the “How” and “Why” questions of achieving pre-determined objectives. The complexity associated with performance evaluations has led to the development of performance measurement systems to collect ongoing data and to monitor and report progress towards pre-determined goals^7,8^.

Performance evaluations addressed through stakeholder questions require that the measures used to report performance results are relevant and trustworthy^10^. A performance ‘outcome’ is defined as the resultant effect of a system towards a pre-determined objective, whereas a performance ‘output’ is the data generated by a single unique metric impacting the outcome^3,11–13^. A balanced performance evaluation includes both internal measurement of the individual units in the program and of the external environment^14,15^. These measurements permit a reflection on achievement to date as well as what *could* be possible to achieve within an equal context^16^. For instance, internal measures of output include ‘exam scores’ in an academic setting, ‘race finish time’ in a sport setting, ‘greenhouse gas emissions per capita’ in an environment setting. Each of these metrics can be used as a performance output measure that represents ‘self-comparison’ when collected over time. However, internal measures alone may be insufficient in performance evaluation as the appraisal of a performance output or trend can vary when assessed relatively against peer performance under comparable circumstances. External environment measures of output include ‘league tables’, ‘event final rankings’ or ‘comparisons with industry benchmarks’ and have become a popular way to compare peers within industries^17–20^. As with internal measures, external measures of relative performance in isolation equally may lack face-validity or attribution (singular allocation) in performance evaluation. This is seen in circumstances where the appraisal of a ranking against peers can vary when interpreted in the context of individual achievement or progress^21–23^.

Examples of bespoke performance measurement systems that comprise singular and multiple output measures exist across industry and sectors: academic^24^, health^25,26^, profit and non-profit organisations^27,28^, sport^29,30^, natural environnent^31,32^, government and private sectors^33–35^, and finance^36,37^. The use of a single output measure to reflect performance outcome may provide a too narrow perspective on achievement in a complex program and therefore risk evaluation face-validity in any of these areas^10^. The selection of performance output measures that enhance attribution and enable meaningful and trustworthy evaluations therefore benefits from appropriate consideration^26,38,39^. Different measurements of performance can be combined into composite scores to increase the face-validity of a program evaluation appraisal without adding difficulty to its interpretation^40,41^. Measurements on different data scales need here to be transformed into a common scale before combining, even though the introduction of re-scaling may reduce reliability^42^. An alignment of scales that reflect a meaningful magnitude change within and between variables can improve face-validity in regards to program outcomes and thereby make a composite score preferred for decision-making^40,43^. It is therefore essential that the measurements are transparent and comprehensible for all stakeholders involved to avoid misleading program decisions using such composite scores^10^. A robust metric must be determined to assess objectively what has occurred and how this may influence future outcomes relative to the investment in any program or individual and any third-party interest.

The aim with this study was to develop a performance scoring template that combines internal and external measures by alignment of scales that reflect meaningful magnitudes of change in stakeholder defined contexts. The purpose is to provide evaluators of applied programs a means to report performance outcomes with convincing face-validity. The scoring template is exemplified by application to evaluation of CO_2_ emissions reduction amongst OECD member countries.

## Results

**Figure 1:**
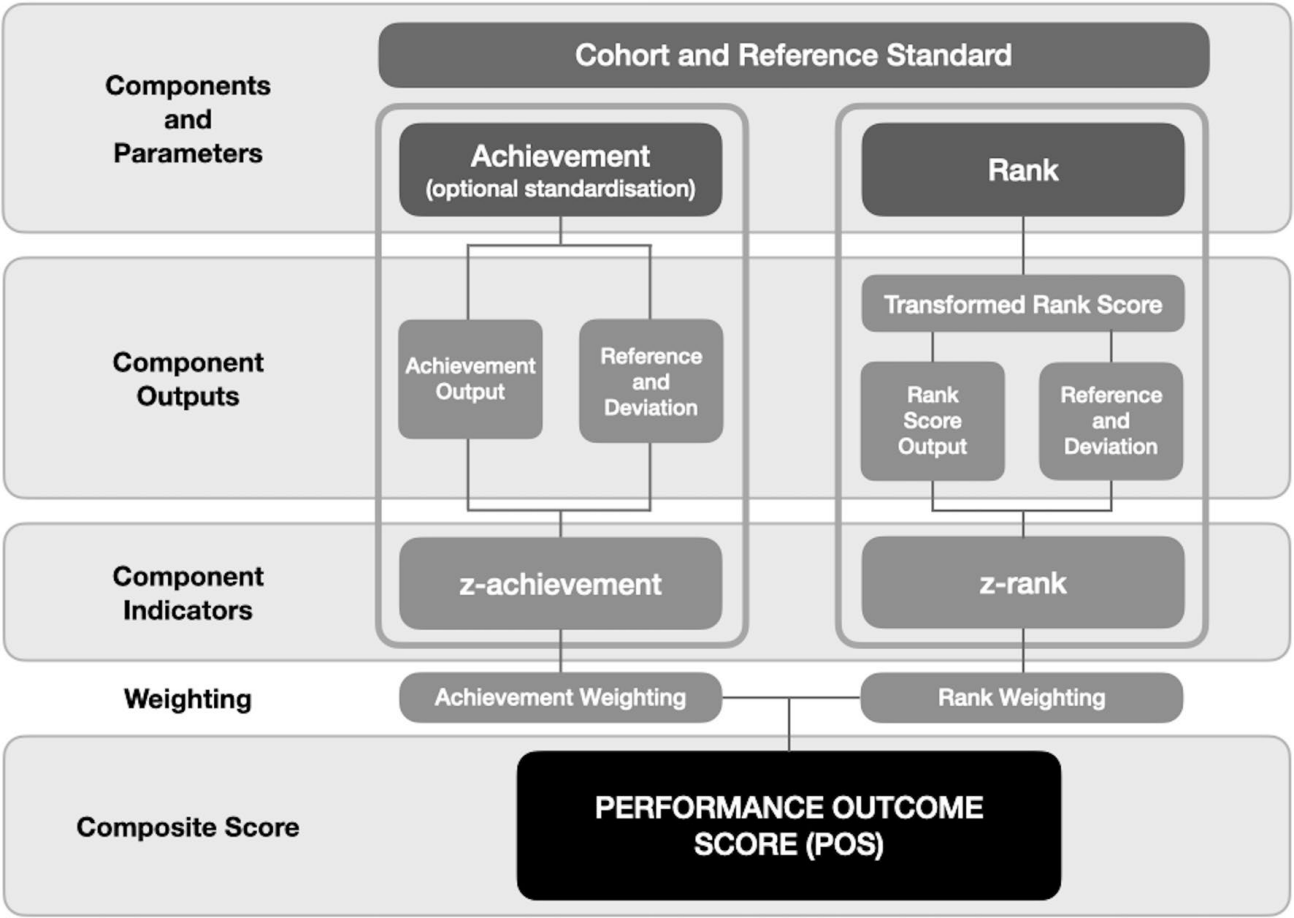
POS-T signifying four cardinal and one optional (weighting) data handling levels.

Application of the performance outcome scoring template (POS-T) commences with selection of data sources and concludes with a composite score that is adjusted for optimal face-validity (POS) (Fig. 1). Subject to the evaluation purpose and selected time-point the template can be utilised in comparing a result with a predetermined objective, benchmark standard or appraise change over time. Statistical software (in the R language) is supplied to support the development of a POS (Data S1). Stepwise instructions provided in the software detail file data set-up for application in the code.

Two generic data sources, achievement (continuous scale) and rank (ordinal scale) are handled in four cardinal and one optional (weighting) index development steps:

- *Components and parameters* Quantifiable domains deemed to have primacy with respect to the face-validity of the performance outcome. Stakeholder selected parameters that frame the evaluation.
- *Component outputs* Performance metrics collected from each component. Representative of the data collection fields that comprise the performance output measure and a comparative reference output within a distribution.
- *Component indicators* Component outputs transformed to normalised measures. The performance outputs measured against a reference standard given assumptions of both achievement and rank deviations.
- *Composite score* A final viable measure is secured through aggregating and optionally weighting normalised component indicators that meet the desired face-validity.

Data handling through the POS-T is described in four detailed steps. Following stakeholder selections made in step 1 the outputs from steps 2–4 are produced automatically when applying the performance data to the statistical R-code software provided (Data S1).

### Step 1: components (quantifiable domains) and parameters

#### Actions

Define evaluation entity (individual, group or population participating in a specified program), sample of entities to evaluate, comparison cohort, and comparator (frame of reference for evaluation). Select quantifiable domains representing internal and external measures that provide face-validity for the performance outcome of an entity (individual or group). Optional selection of a standardising parameter.

#### Outcome

Defined entity(s), cohort, comparator, and quantifiable domains with arguments for their selection. Optional standardising parameter.

#### Procedure

The entities for evaluation are selected within a cohort of interest framed by the context of the comparator. Examples of evaluation comparators include referencing a previous time point to evaluate performance over time, referencing a population mean to evaluate performance of the entity against a population standard, or referencing a single measure like a season average or predetermined objective to evaluate the entity against expectation. Next is selecting quantifiable domains that represent the *components* of ‘achievement’ (measured on a continuous scale) and ‘rank’ (measured on an ordinal scale). For the entity being evaluated ‘achievement’ is characterised as the component denoting self-comparison (internal measure), and ‘rank’ characterises the component denoting comparison with others (external measure). The optional selection of a standardising parameter is applied to the continuous data component. Standardising parameters are measures of exposure applied to the continuous data component and examples include: ‘per capita’ calculations, standardisation by funding, access to resources, or other parameters of exposure. Completion of step 1 is made by recording the arguments behind selecting each of the components and describing the cohort parameters, reference standards and optional standardising parameters.

### Step 2: component outputs (performance metrics from each component)

#### Action

Collect/calculate performance metrics from each component.

#### Outcome

For achievement and rank performance metrics refer to: Output, Reference, and Deviation.

#### Procedure

Performance metrics are recorded for both achievement and rank. The ‘achievement output’ and ‘rank score output’ are established as well as descriptive data for each component parameter, i.e. references and deviations (Table 1). This is completed for each ‘entity’ (individuals or groups) in the cohort. When referring to separate entity’s subscripts ‘*i*’ and ‘*j*’ are used.

**Table 1:** Component parameters and how they are quantified for an entity ‘*i*’.

Component	Component output	Metric quantification description
Achievement	Achievement output (*O*_*A*,*i*_)	Outcome of interest crude measure
	Achievement reference (*R*_*A*,*j*_)	Achievement output from previous time-point
		OR
		Mean achievement output over a time-period
		OR
		A population standard
		OR
		Other comparator of interest measured by the same metric and continuous scale
	Achievement deviation (*D*_*A*,*i*_)	Deviation of:
		Individual achievement outputs over a time-period
		OR
		Population achievement outputs from peers in cohort of interest
		OR
		Population deviation for metric of interest
	Lower and upper reference limiters	Practical lower and upper reference limits. To establish a cohort range for consistent future comparative evaluations
Rank	Final rank (*R*_*i*_)	Entity rank in order of crude measures at evaluation time-point
	Initial rank (*ρ*_*i*_)	Entity rank at time-point of comparison prior to evaluation event or period
	Transformed rank-scores $f\left( {\rho_{i} } \right)$	Ranks transformed to a continuous value. Reflecting non-equidistance between entity ranks generated from crude measure
	Rank score output $\left( {O_{RS,i} } \right)$	Magnitude difference of final rank position relative to peers. Aggregation of transformed rank-scores from entities with a final rank behind entity $i$
	Rank score reference $\left( {R_{RS,i} } \right)$	Median of simulated measures $\Phi_{i}$ for each entity
	Rank score deviation $\left( {D_{RS,i} } \right)$	Deviations of simulated rank score outputs. Absolute value of the difference between the median and either of *P*_16_ or *P*_84_ of simulated measures $\Phi_{i}$ for each entity

#### Achievement performance metrics

##### Output

The ‘*achievement output*’ (*O*_*A*_) is quantified directly from the metric of interest crude measure (continuous scale) and reflects the output *being evaluated*.

##### Reference

The ‘*achievement reference*’ (*R*_*A*_) is the metric of interest crude measure from a previous time point and is used as the metric of comparison as framed by the comparator defined in step 1. The achievement reference and output are measured by the same metric on the same continuous scale.

##### Deviation

The ‘*achievement deviation*’ (*D*_*A*_) is the deviation of crude measures across the observation period or other collection of entity crude measures that constitute a deviation around the achievement reference. The reference and deviation values are set from the decision to use a cohort pooled achievement standard deviation or individual entity standard deviations. The achievement outputs are assumed to follow a normal distribution, $N\left( {\overline{{A_{i} }} ,\sigma_{{A_{i} }} } \right)$, from which the reference and deviation values are set; *R*_*A*,*i*_ = $\overline{{A_{i} }}$ and *D*_*A*,*i*_ = $\sigma_{{A_{i} }}$.

#### Rank performance metrics

**Figure 2:**
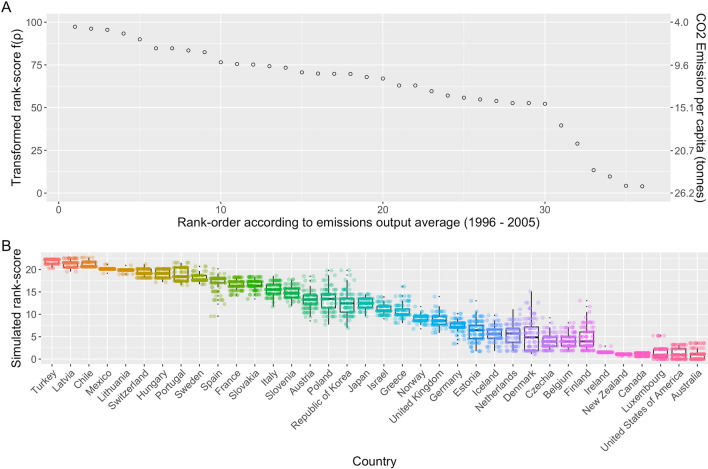
(**A**) Entity rankings, (**B**) Simulated rank scores (example data). (**A**) Initial rank-order (ranked by average annual CO_2_ emissions per capita 1996–2005) (ρ_i_) versus average CO_2_ emission per capita 1996–2005 (tonnes) and transformed rank-score f(ρ). (**B**) Simulation (example with 100 iterations shown for illustrative purposes) of potential rank score outputs (Φ_*i*_) for each entity based on underlying distribution of achievement output. Box plot showing 16th and 84th percentiles.

Rank scores (forming the ordinal ranking order) are transformed to a continuous scale value (transformed rank-score) using a pre-defined function, *f*, to reflect non-equidistance (magnitude difference) between different entities based on the continuous metric that established the ranking order. Lower and upper ‘reference limiters’ are applied to establish the transformed rank-score range. The reference limiters represent the range of minimum and maximum reference scores and establishes a range for future equivalent comparisons. Selection should consider a range beyond current reference score minima and maxima that would account for a realistic range of future reference score possibilities. The lower achievement limiter is attributed a transformed rank-score of 1 and the upper achievement limiter is attributed a transformed rank-score of 100 (Fig. 2A). In different program settings either a higher or lower achievement score may reflect the ‘best’ performance. This directionality is established during the stakeholder selections at the start of the statistical R-code data handling process.

##### Output

The ‘*rank score output*’ (*O*_*RS*_) is formed by, for each *i*, aggregating the comparison of transformed rank-scores between entity *i*, $f\left( {\rho_{i} } \right),$ and each other entity *j*, $f\left( {\rho_{j} } \right)$, when entity *i* has a better final rank ($R_{i}$) relative to the final rank of entity *j* ($R_{j}$). This reflects meaningful magnitude differences between the final ranks for entities *i* and *j*.

For entity *i* the ‘rank score output’ (*O*_*RS*,*i*_) is then defined as:

1 $$O_{RS,i} = \mathop \sum \limits_{\forall j \ne i} \delta \left( {i,j} \right)$$

where

2 $$\delta \left( {i,j} \right) = \left\{ {\begin{array}{*{20}l} {\frac{{f\left( {\rho_{j} } \right)}}{{f\left( {\rho_{i} } \right)}},} \hfill & {\quad if\;R_{i} < R_{j} } \hfill \\ {0,} \hfill & {\quad if\;R_{i} \ge R_{j} } \hfill \\ \end{array} } \right.$$

##### Reference and deviation

The underlying performance metrics for ‘rank score outputs’ are generated using a *simulator*. The simulator generates an underlying distribution of rank score outputs based on the underlying distribution of possible achievement outputs. It randomly selects one achievement output for each entity, transforms them into ranks, transformed rank-scores, and finally rank score outputs. The outputs are saved for each entity *i* and the procedure is iteratively repeated, resulting in distributions of probable rank-score outputs, Φ_*i*_, for each entity *i* (Fig. 2B).

For each entity *i*, the ‘*rank score reference*’, *R*_*RS*,*i*_, and the ‘*rank score deviation*’, *D*_*RS*,*i*_, are chosen as:

3 $$R_{RS,i} = Median\left( {\Phi_{i} } \right)$$

4 $$D_{RS,i} = \left\{ {\begin{array}{*{20}l} {Median\left( {\Phi_{i} } \right) - P_{16} \left( {\Phi_{i} } \right),} \hfill & {\quad if\; O_{R,i} < Median\left( {\Phi_{i} } \right)} \hfill \\ {P_{84} \left( {\Phi_{i} } \right) - Median\left( {\Phi_{i} } \right),} \hfill & {\quad if\; O_{R,i} \ge Median\left( {\Phi_{i} } \right)} \hfill \\ \end{array} } \right.$$

where P_16_ and P_84_ are the 16th and 84th percentiles, respectively.

### Step 3: component indicators (normalised component outputs)

#### Action

Component outputs transformed to normalised measures reflecting the magnitude change in achievement and rank.

#### Outcome

Z-achievement and z-rank.

#### Procedure

Normalised *z*-scores are calculated based on the component outputs. Z-achievement and z-rank indicators are established as proportional measures of output deviation from their corresponding references.

For the achievement component:

5 $$z_{A,i} = \frac{{signum \cdot \left( {O_{A,i} - R_{A,i} } \right)}}{{D_{A,i} }}$$

where

$$signum = \left\{ {\begin{array}{*{20}l} {1, } \hfill & {\quad if\;lower\;achievement\;scores\;are\;better} \hfill \\ { - 1,} \hfill & {\quad if\;higher\;achievement\;scores\;are\;better} \hfill \\ \end{array} } \right.$$

For the rank component:

6 $$z_{RS,i} = \frac{{O_{RS,i} - R_{RS,i} }}{{D_{RS,i} }}$$

### Step 4: composite score

#### Action

Aggregated component indicators with optional weighting.

#### Outcome

Performance outcome score (POS).

#### Procedure

The component indicators are combined in a weighting procedure based upon a user pre-defined setup of their respective relevance. An argument for the choice of weights for each respective component is formulated. The POS is established together with an explanation of the component weighting. When applying the option of proportional weighting to the component indicators the larger the importance of the component, the higher the weighting factor: *w*_*A*_ for achievement and *w*_*R*_ for rank. The weighting is performed on centred z-scores for achievement $c_{A,i}$ and rank $c_{R,i}$ respectively:

7 $$c_{A,i} = z_{A,i} - M_{A}$$

8 $$c_{R,i} = z_{R,i} - M_{R}$$

where

9 $$M_{A} = \mathop {{\text{mean}}}\limits_{\forall i} \left( {z_{A,i} } \right)$$

10 $$M_{R} = \mathop {{\text{mean}}}\limits_{\forall i} \left( {z_{RS,i} } \right)$$

After the weighting, the score is shifted back so that the centre of the composite score reflects the centre of the achievement indicator, so, the composite score for *entity* $i$ is then defined as

11 $$\begin{aligned} POS_{i} & = \frac{{w_{A} c_{A,i} + w_{R} c_{R,i} }}{{w_{A} + w_{R} }}+M_{A} \\ & = \frac{{w_{A} \left( {Z_{A,i} - \mathop {{\text{mean}}}\limits_{\forall i} \left( {Z_{A,i} } \right)} \right) + w_{R} \left( {Z_{R,i} - \mathop {{\text{mean}}}\limits_{\forall i} \left( {Z_{R,i} } \right)} \right)}}{{w_{A} + w_{R} }} + \mathop {{\text{mean}}}\limits_{\forall i} (z_{A,i} ) \\ \end{aligned}$$

#### POS-T application example

The context is 36 OECD countries (excluding countries with incomplete data publicly available) that have set out to reduce their CO_2_ emissions over the 10-year period 2006–2015 in the global reduction of greenhouse effect (Table 2). Four entities (Sweden, Mexico, Norway, and Luxembourg) were hypothetically to be evaluated with regard to three performance goals:

**Table 2:** Contextual features framing the evaluation.

Context	Description
Entities to evaluate	Four countries (Sweden, Mexico, Norway, Luxembourg)
Comparison cohort	36 OECD countries
Comparator	CO_2_ emissions per capita over the period 2006–2015
Quantifiable domains (components)	Internal comparison—achievement
	External comparison—rank

Output goals:

Goal 1. “To evaluate reduction in CO_2_ emissions per capita over the period 2006–2015”.

Goal 2. “To evaluate change in international ranking with respect to CO_2_ emissions per capita”.

Outcome goal:

Goal 3. “To evaluate reduction performance relative to peers regarding CO_2_ emissions per capita over the period 2006–2015”.

To develop performance outcome evaluation measures for these goals using the POS-T, the comparative evaluation cohort is first described. The four countries (entities) will have their performance evaluated against the cohort of 36 OECD countries. The comparator framing the evaluation was defined as a comparison of performance over time.

### Example step 1: components (quantifiable domains) and parameters

Tonnes of CO_2_ equivalent (“CO_2_ emissions”) was chosen as the achievement output measure for the evaluation of Goal 1 (Table 3). A standardising parameter of ‘per capita’ was applied to the achievement component to permit direct comparison between cohort countries on a per capita basis. The achievement reference was defined as the average annual CO_2_ emissions per capita during the 10-year period 1996–2005 and the achievement output for comparison was defined as CO_2_ emissions per capita in the year 2015. The OECD world ranking table position was chosen as the rank output measure when evaluating Goal 2. To evaluate Goal 3, the POS-T was used to develop a POS that depicts the performance outcome regarding emission change over time for evaluation in the context of self-comparison and comparison with peers.

**Table 3:** Components, parameters, and metrics used to populate the component outputs.

Component	Parameter	Metric
Achievement	Output measure	Tonnes of CO_2_ equivalent 2015
	Standardising parameter	per capita
	Reference	10-year annual output average 1996–2005
	Distribution	Individual entity annual variations 1996–2005
Rank	Output measure	OECD ranking table 2015
	Reference	Rank of median achievement references

### Example step 2: component outputs (performance metrics from each component)

Data were managed following the stepwise process detailed in the R-code software provided (Data S1). Data for each component were collected^44^ and presented in file format (Data S2 and S3). Lower and upper reference limiters were set beyond the minimum and maximum CO_2_ emissions per capita outputs. Individual entity standard deviations were chosen for use in the simulations. Application of the POS-T R-code to the performance data produced automated performance outputs and descriptive statistics. The following component outputs were generated (Fig. 2B and Table 4).

**Table 4:** Component outputs for the four stakeholders derived from Step 2 of the POS-T. (Units of achievement = tonnes of CO_2_ equivalent per capita. Rank score output = proportional score gained from ranking ahead of other countries. Rank score reference = median score from simulation based on achievement descriptive statistics. Rank score deviation = simulation outputs based on 16th and 84th percentiles. LUX. = Luxembourg).

Component	Component output	Sweden	Mexico	Norway	Lux
Achievement	Achievement output (*O*_*A*,*i*_)	5.47	5.74	10.47	18.17
	Achievement reference (*R*_*A*,*j*_)	7.90	5.48	12.23	24.06
	Achievement deviation (*D*_*A*,*j*_)	0.34	0.10	0.14	2.78
	Lower and upper reference limiters	Lower reference limiter 4 Upper reference limiter 26			
Rank	Final rank (*R*_*i*_)	1	3	24	33
	Initial rank (*ρ*_*i*_)	9	4	21	34
	Transformed rank-score out of 100 $\left( {f\left( {\rho_{i} } \right)} \right)$	82.44	93.36	62.97	9.72
	Rank score output $\left( {O_{RS,i} } \right)$	26.44	21.32	7.14	2.23
	Rank score reference $\left( {R_{RS,i} } \right)$	17.65	20.14	8.61	0.86
	Rank score deviation $\left( {D_{RS,i} } \right)$	1.01 (16.63–18.66)	1.28 (18.86–21.42)	0.05 (8.56–8.66)	1.38 (− 0.53 to 2.27)

Goal 1. “To evaluate reduction in CO_2_ emissions per capita over the period 2006–2015”.

Component output: Three of the four countries reduced their raw CO_2_ emissions per capita. Luxembourg saw the largest reduction of the four example countries and largest reduction compared to the full OECD cohort (− 5.90 tonnes per capita), followed by Sweden (13th overall; − 2.43 tonnes per capita), and Norway (21st overall; − 1.76 tonnes per capita). Mexico saw an increase in CO_2_ emissions (30th overall; + 0.26 tonnes per capita).

Goal 2. “To evaluate change in international ranking with respect to CO_2_ emissions per capita over the period 2006–2015.

Component output: Three of the four countries improved their ranking. Sweden (= 3rd largest rank shift overall: + 8 places), Luxembourg and Mexico (= 10th largest rank shift overall: + 1 place) relative to all 36 comparison countries. Norway fell in ranking (= 27th largest rank shift overall: − 3 places) reflecting having not reduced their emissions per capita to the same level over the observation period as the comparison cohort.

### Example step 3: component indicators (normalised component outputs)

The component outputs were transformed to normalised measures demonstrating internal and external magnitude change relative to the achievement and rank reference standards respectively (Table 5). The three countries that saw a reduction in raw CO_2_ emissions per capita demonstrated positive internal magnitude change (z-achievement: Sweden: + 6.85, Norway: + 12.92, Luxembourg: + 2.12). One country saw an increase in raw CO_2_ emissions per capita demonstrating negative internal magnitude change (z-achievement: Mexico: − 2.56). Three of four countries improved their ranking demonstrating positive external magnitude change (z-rank: Sweden: + 8.69, Mexico: + 0.92, and Luxembourg: + 1.00). One country regressed in ranking demonstrating a negative external magnitude change (Norway: − 27.73).

**Table 5:** Component indicators (normalised component outputs) derived from Step 3 of the POS-T.

Indicator	Sweden	Mexico	Norway	Luxembourg
z-achievement $\left( {z_{A,i} } \right)$	6.85	− 2.56	12.92	2.12
z-rank $\left( {z_{RS,i} } \right)$	8.69	0.92	− 27.73	1.00

### Example step 4: composite score (POS)

The selected weighting ratio for achievement and rank was set at 1:1. The relative magnitude of change (component indicators) for both component outputs were combined, resulting in a composite score (POS) (Table 6).

**Table 6:**
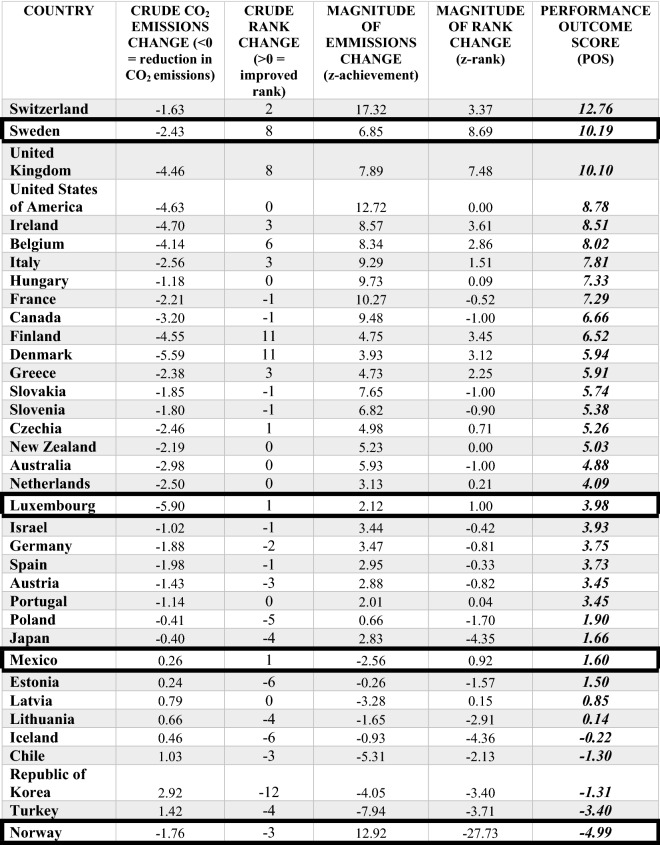
OECD countries ordered highest to lowest by the POS including component crude output variation and component magnitude of change over the observation period. (Crude CO_2_ emissions = tonnes of CO_2_ equivalent per capita).

Significant values are in bold and italics.

Outcome goal:

Goal 3: “To evaluate reduction performance relative to peers regarding CO_2_ emissions per capita over the period 2006–2015”.

Composite score: The combined relative magnitude of change for the component indicators showed a positive development of performance for Sweden (+ 10.19), Luxembourg (+ 3.98), and Mexico (+ 1.60), and a negative development of performance for Norway (− 4.99).

### Summary information gained for performance evaluation (4 stakeholder countries highlighted)

See Table 6.

## Discussion

This study set out to develop a scoring template that combines internal and external measures of performance by alignment of measurement scales which represent meaningful magnitudes of change. The resulting POS-T adheres to the principle of providing the stakeholders governing applied programs a means to report performance outcomes with convincing face-validity^39,45,46^. We exemplified application of POS-T in an evaluation of CO_2_ emission reduction amongst OECD member countries. Flexible and transparent evaluation methods oriented towards stakeholders and usefulness have repeatedly been asked for in the environmental sciences^47,48^. To ensure that POS-T produces scores useful for stakeholders, an inductive (discovery) approach was found best suited^49,50^. This approach aligns with the principles of design thinking^51^ where the emphasis is placed on defining the problem to be solved through the needs of the stakeholders involved^45,46^. In the following, the main features of the POS-T are discussed considering the CO_2_ emission example and directions are outlined for future research.

The measures and methods available to report performance results delimit a stakeholder’s capacity to evaluate applied programs^40^. In their governance, accomplishment has been described as the gap between expected and actual output or the deviation of the output from an industry standard^12^. The POS-T provides in Step 2 methodology to establish a *magnitude* of this gap for both the achievement and rank components, i.e. for internal and external comparisons. In step 3, ready-to-combine component indicators are formed by normalisation of the component outputs produced. The resulting component indicators describe magnitudes of change, i.e. the gaps between expected and actual results or change over time for internal and external comparisons. Producing the relative magnitude of change from a point of reference for both components (achievement and rank) rather than binary measures alone provides those evaluating the outcome with greater context. For example, on a binary scale, Sweden, Luxembourg, and Norway each demonstrated a *reduction* in CO_2_ emissions per capita (positive achievement outcome) relative to their own reference standard in 2005. However, when accounting for the external context, Norway during the evaluation period slipped down the ranking table from 21st to 24th (negative rank outcome) due to that other countries reduced their relative emissions by greater amounts. Conversely, Mexico gained a ranking place from 4th to 3rd (positive rank outcome) even having had a small increase in crude CO_2_ emissions due to that other countries close in rank had relatively larger increases in crude CO_2_ emissions. Proportional output measures relative to self-comparison and comparison with others in a chosen cohort provides a context for stakeholders to better frame and evaluate an outcome against expectation and describe the overall accomplishment.

Integration of self-comparison and rank change magnitudes adds complexity to program evaluation indicators. Maintenance of face-validity in such composite scores requires measurement system transparency^52^. The POS-T supports transparency and face-validity by offering evaluators semantic clarity regarding the components of the integrated composite score. Stakeholders evaluating performance using the POS-T will base their assessments on normalised indicators of any measured or pre-existing method of reporting achievement output for internal (within-individual) comparisons. The crude achievement outputs can in any circumstance be ranked^53^ and the normalised indicators computed for rank changes and external comparisons (between individuals). The normalised indicators are calculated in a standardised manner, i.e. for ‘achievement’ by subtracting the mean from an individual raw score and then dividing the difference by the standard deviation. The mean and standard deviation are based on individual, or population standards as chosen in step 1 by the stakeholder evaluating the performance. Normalised indicators are calculated for ‘rank’ by transforming the ordinal scale to continuous relative values before applying the same normalisation process to a series of simulated rank outputs. Such normalised indicators are well-known and are broadly used in, for instance, global health settings for comparative evaluations of development processes at individual and population levels, e.g. in the child growth area^54,55^. In the example application of POS-T on CO_2_ emissions, the normalised ‘self-comparison’ indicator showcases that Luxembourg’s crude emissions reduction from the reference of 2005 is achieved in the context of a broader distribution of the annual fluctuation in emissions by Luxembourg compared to Sweden. In essence, Sweden’s emissions reduction achievement is at face value more substantial in the context of self-comparison due to the narrower distribution of annual emissions fluctuations. The magnitude of this achievement is demonstrated by a higher achievement indicator. Regarding external comparisons, both Luxembourg and Mexico gained one place on the ranking table, yet the normalised rank indicator calculated in step 3 shows that the magnitude of Luxembourg’s gain is greater than Mexico’s. This is due to that Mexico’s emissions per capita were very close in volume to those countries with similar rank, the effect being that a change in rank may occur even from small changes in emissions output resulting in a smaller rank indicator for the same crude rank change. By using the continuous data that formulates the ranking order, context and magnitude is apportioned to the component indicator in step 3 representing the rank change. Internal and external measures presented as a magnitude of change against a reference and accompanying distribution provide meaningful context to the performance. The selection of the lower and upper reference limiters provides an important step when applying the statistical code in establishing consistent comparators and context for future equivalent evaluations. Performance outcomes presented this way can be used to observe longitudinal performance trends within an individual entity or relative to a population as well as measuring a single performance against expectation.

Some limitations to the use of the POS-T are important to consider. In experimental evaluation research, influence from external factors is controlled in the study design. Emulating an experimental design in observational performance evaluations in practice settings would require information on all confounding factors^56^. Application of the POS-T does not per se assure that the POS reflects causal effects of the program, and consideration of confounding factors is always needed when interpreting POS scores in practice settings. Moreover, it should be taken into regard that the simulation process used in POS-T to determine the rank score deviations for each entity uses the reference and its standard deviation as assumptions in the calculation. The outcome from each simulation may thus vary slightly. This effect is minimised by always running an adequate number of simulations on each occasion. Furthermore, when the data available to calculate the achievement deviation is limited, a decision must be made regarding what to use as the achievement deviation for comparison with the achievement output. The preferred option is to use the achievement deviation unique to each entity. However, an option is to use the cohort population standard deviation as this broadens the dataset to calculate deviations and improves its reliability. This may be a satisfactory solution when the comparative data sets between entities in the cohort have similar deviations. If this is not the case, an option may be to use the largest or smallest deviation in the cohort. The flexibility in selecting components, standardising parameters and weighting of the component indicators opens the composite score to variability in its robustness. Testing for robustness is recommended and aided by the level of transparency described by the evaluator in selecting optional features in corresponding steps of the POS-T framework.

The POS-T in its current form can be applied to any program governance setting. A POS can be determined for single entities at multiple time points to assess performance trends or for multiple entities at a single time point to assess performances relative to a population standard or to peers. The component indicators can also be used to evaluate each component in isolation. The rank simulation process in isolation may furthermore be utilised to determine probabilities of performance outcome. In a sports context, where both personal achievement and comparative rank are considered in a performance evaluation, the POS-T may provide valid comparison of performance outcomes by individual athletes across the span of a career. In this example the evaluator has flexibility in selecting the achievement reference, e.g. a population standard, or the athletes own season averages. The POS-T can also provide a point in time comparison between individual competitors within an event or funded individual sports programs within a country. In an education context the POS-T may be applied to the performance evaluation of students across semesters or to evaluate the performance of education institutions over time wherever a ranking score is calculated and ranking table produced. The POS-T can be applied to any evaluation setting where a continuous data metric could be used to rank entities. Further development of the POS-T will include development of the statistical code to include the evaluation of performance in settings where achievement is not readily quantified, e.g. when it mainly is established through head-to-head contests.

## Conclusion

The POS-T endorses face-validity in real-world program evaluations by that the resulting POS reflects a meaningful magnitude of performance outcome with regards to self-comparison and comparison with peers. The template is presented with statistical software for creating scoring systems and is exemplified by evaluation of CO_2_ emissions reduction amongst 36 OECD member countries. Forthcoming research will involve application of the POS in different applied performance evaluation settings.

## Materials and methods

### Construction principles

**Figure 3:**
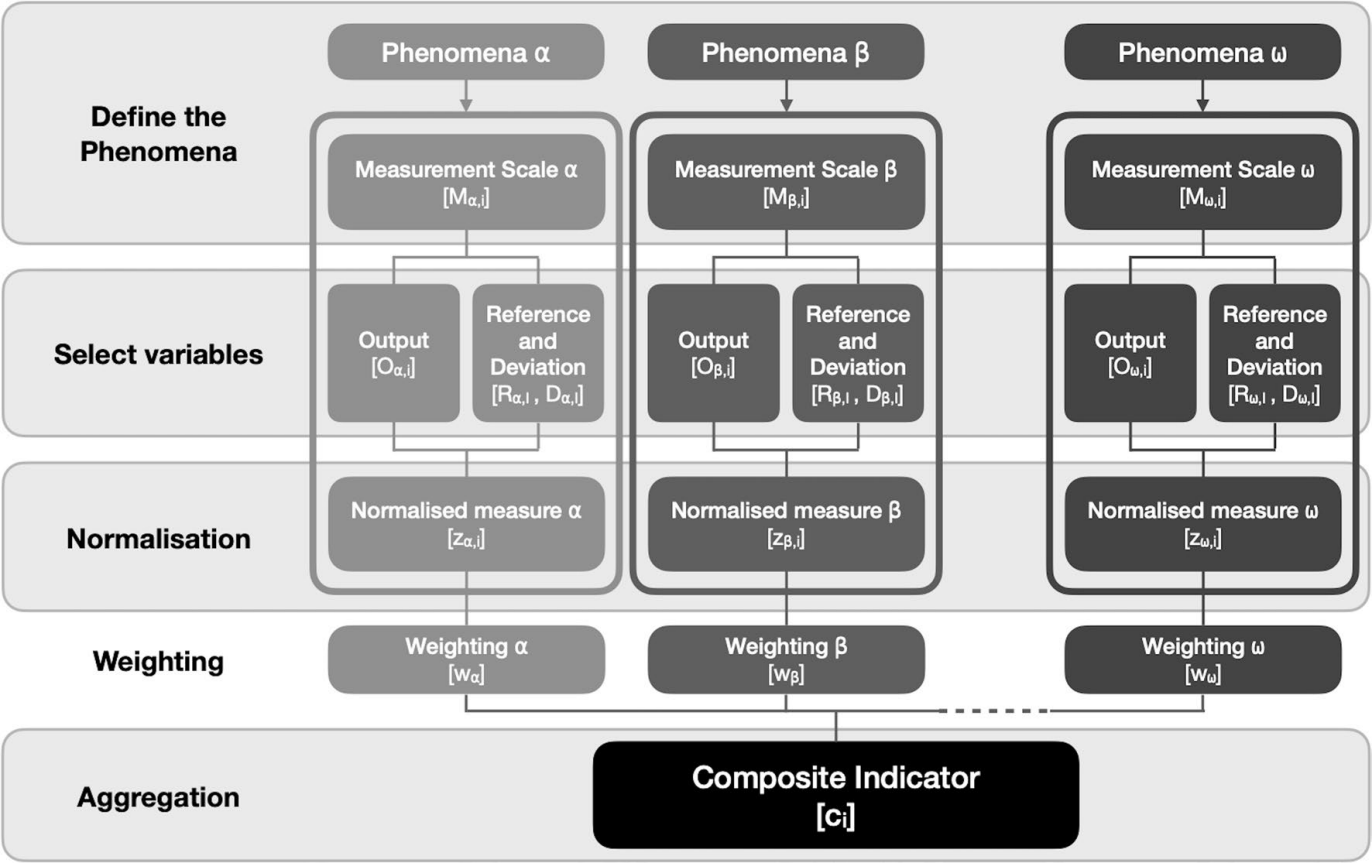
Composite score development model with five development steps and three example phenomena.

Construction of the POS-T employed an iterative approach to solution design that prioritises application of the final template in real-world program governance settings. Its practical use was further supported through the parallel construction of a statistical framework and software for score development in applications^50^. A design panel was composed for the construction consisting of scientists and practitioners (n = 5) with backgrounds in epidemiology, public health and sport settings, organisational development, statistical methods, and experimental design. A composite score development model was used to guide the construction process (Fig. 3).

### Template construction process

The design panel met via an online meeting platform weekly over a twelve-month period and discussed the POS-T development in the context of four cardinal steps and one optional step depicted in the construction model (Fig. 3). In each development model step, the design panel employed an iterative process applying varying methods of data analysis and representation in the template to identify potential inconsistencies or errors in the composite score. These were identified, discussed, and addressed at each online meeting until panel consensus on user application was reached. Consensus required agreement on the stepwise process necessary for the user when defining the context of POS-T evaluation. Once the process was established and incorporated into the POS-T consensus on the maintenance of the aggregated composite score face-validity was obligatory.

Statistical code was written using R programming language to automate the methodological outputs of steps 2–4 (Data S1). Data comprising the variables outlined in step 2 of the development model were systematically organised in data files using Microsoft Excel 2016 (Data S2 and S3). The current version of the R-code was written to use with discrete cohorts that comprise all entities in sequential ranking order for analysis.

### POS-T application example

The resulting POS-T was finally applied in an evaluation of reduction of greenhouse gas emission among 36 OECD countries. The OECD has collected and reported annual greenhouse gas emissions between 1996 and 2015 for a cohort of 36 countries producing an annual emissions ranking table^43^. The OECD data set was used to showcase performance evaluations based on a composite score for comparison within either of the singular reporting metrics; tonnes of CO_2_ emissions equivalent per capita, or the emissions ranking table. To showcase the template and the statistical framework, the design panel took on the virtual role of an international stakeholder commission in the environmental protection area. The final statistical framework was exemplified by applying the R-code to the 36 country OECD data set and analysing the performance outcomes of four countries: Sweden, Luxembourg, Mexico, and Norway.

## Supplementary Information

Supplementary Information 1.

Supplementary Information 2.

Supplementary Information 3.

## Data Availability

All data are available in the main text or the supplementary materials.
